# Plasma N-glycome shows continuous deterioration as the diagnosis of insulin resistance approaches

**DOI:** 10.1136/bmjdrc-2021-002263

**Published:** 2021-09-09

**Authors:** Ana Cvetko, Massimo Mangino, Marko Tijardović, Domagoj Kifer, Mario Falchi, Toma Keser, Markus Perola, Tim D Spector, Gordan Lauc, Cristina Menni, Olga Gornik

**Affiliations:** 1 University of Zagreb Faculty of Pharmacy and Biochemistry, Zagreb, Croatia; 2 Department of Twin Research and Genetic Epidemiology, King’s College London, London, UK; 3 NIHR Biomedical Research Centre at Guy’s and St Thomas’ Foundation Trust, London, UK; 4 National Institute for Health and Welfare, Helsinki, Finland; 5 Genos Glycoscience Research Laboratory, Zagreb, Croatia

**Keywords:** insulin resistance, glycosylation, diabetes mellitus, type 2, preventive medicine

## Abstract

**Introduction:**

Prediction of type 2 diabetes mellitus (T2DM) and its preceding factors, such as insulin resistance (IR), is of great importance as it may allow delay or prevention of onset of the disease. Plasma protein N-glycome has emerged as a promising predictive biomarker. In a prospective longitudinal study, we included patients with a first diagnosis of impaired glucose metabolism (IR or T2DM) to investigate the N-glycosylation’s predictive value years before diabetes development.

**Research design and methods:**

Plasma protein N-glycome was profiled by hydrophilic interaction ultra-performance liquid chromatography in 534 TwinsUK participants free from disease at baseline. This included 89 participants with incident diagnosis of IR or T2DM during the follow-up period (7.14±3.04 years) whose last sample prior to diagnosis was compared using general linear regression with 445 age-matched unrelated controls. Findings were replicated in an independent cohort. Changes in N-glycome have also been presented in connection with time to diagnosis.

**Results:**

Eight groups of plasma N-glycans were different between incident IR or T2DM cases and controls (p<0.05) after adjusting for multiple testing using Benjamini-Hochberg correction. These differences were noticeable up to 10 years prior to diagnosis and are changing continuously as becoming more expressed toward the diagnosis. The prediction model was built using significant glycan traits, displaying a discriminative performance with an area under the receiver operating characteristic curve of 0.77.

**Conclusions:**

In addition to previous studies, we showed the diagnostic potential of plasma N-glycome in the prediction of both IR and T2DM development years before the clinical manifestation and indicated the continuous deterioration of N-glycome toward the diagnosis.

**Significance of this study**

**What is already known about this subject?**

- Type 2 diabetes mellitus (T2DM) and its risk factors represent a huge public health burden and identifying biomarkers that can predict disease onset would allow prevention or delay of onset of T2DM.
- There is a wide array of available T2DM prediction models, many of them are relying on pathophysiological changes of common diabetes biomarkers, which are usually a consequence of an already developed metabolic disorder and are therefore often unable to identify at-risk individuals early enough in the disease development process.

**What are the new findings?**

- In this study, most of our participants had insulin resistance (IR) as their first confirmed diagnosis of impaired glucose metabolism, and since IR is known as a condition which very commonly precedes the development of T2DM for even a decade, we have now evaluated N-glycans as a predictive biomarker even earlier in life.
- Our results also indicated the continuous deterioration of N-glycome toward the diagnosis of impaired glucose metabolism.
- Our findings point out the importance of using plasma N-glycans in existing and future T2DM prediction models and risk assessment tools since we have shown that glycans encompass the information body mass index data carry, but also provide additional predictive value.

**How might these results change the focus of research or clinical practice?**

- It is still unknown whether these changes are the cause or the consequence of the disease; however, it is now certain that they play an important role in the diabetes development and that the change of glycans within a person could warn about possible disease development and allow both the clinician and the patient to take adequate steps to prevent or delay disease development.

## Introduction

Type 2 diabetes mellitus (T2DM) is one of the most prevalent metabolic diseases in the world, with over 400 million individuals living affected with this chronic disease.^1 2^ With multiple cardiovascular, metabolic and even neurological comorbidities developing alongside T2DM,^3^ the search for effective medications and therapies would be highly beneficial both for the drug-developing companies and the patients.^4^ However, T2DM is a chronic, ongoing, incurable disease^5 6^ held under control by medication, but only to prevent further development of comorbidities and to resolve symptoms. Even though some studies presented the possibility of reversing T2DM, such claims are still not clinically proven.^7 8^

Thus, type 2 diabetes and its risk factors represent a huge public health burden and identifying biomarkers that can predict disease onset would allow prevention or delay of onset of T2DM. Early T2DM prediction was mostly based on the recognition of insulin resistance (IR) in the pre-diabetic state.

IR, defined as the inability of insulin to increase glucose uptake and usage in an individual as much as in healthy population,^9^ is a long-known major risk factor for the development of T2DM.^10^ IR accompanied with abnormally increased body fat is a hallmark of pre-diabetes, which has recently been noted as a highly probable major underlying condition for the development of metabolic syndrome.^11^ Moreover, pre-diabetes accompanied with IR and deteriorated β cells accounts for approximately 5%–10% of patients with newly diagnosed T2DM per year.^12^

Many studies investigated both basic IR/T2DM prediction models using standard, accessible variables like blood glucose or insulin levels, as well as improved models upgraded with additional biomarker or body measurement data. A review article from 2012^13^ combined multiple diabetes prediction models, basic or improved, in order to assess their predictive and discriminative performance. The authors concluded that the basic models performed well on their own, but additional biomarker data made extended models accomplish better results. The following general worldwide progress of technology, computational models and statistical analyses profusely impacted the field of diabetes prediction by using data mining and machine learning to extensively upgrade and improve prediction tools and software.^14–16^ Even though there is a wide array of available T2DM prediction models, many of them are relying on pathophysiological changes of common diabetes biomarkers, which are usually a consequence of an already developed metabolic disorder and are therefore often unable to identify at-risk individuals early enough in the disease development process. Taking this into account, there is still a great need for T2DM predictive biomarkers, such as N-glycans, that could identify individuals at the very start of their metabolic deterioration, while they still appear as healthy.

N-glycans are oligosaccharide structures added to the polypeptide sequence of a protein via the enzymatically mediated and highly regulated process of N-glycosylation.^17^ N-glycosylation, a process regulated by a complex network of genes,^18^ is different from glycation, a non-enzymatic addition of sugars to proteins.^19^ N-glycosylation is among the most prevalent co-translational and post-translational modifications, with the vast majority of eukaryotic proteins being glycosylated.^17^ Since it is known that post-translational modifications greatly impact structural and functional features of proteins,^20^ it comes as no surprise that N-glycans were examined in the many physiological and pathophysiological conditions caused by protein diversification, in which they showed to be significant.^21 22^ Recent studies have proposed the idea of glycans as functional effectors in various physiological processes, as well as different disorders and diseases, such as diabetes and obesity-induced IR.^23–26^ Importantly, mostly proinflammatory-like changes of both plasma and IgG N-glycome were observed in two separate cross-sectional studies of patients with T2DM.^27 28^ Plasma N-glycome was also identified to have great discriminative power for other types of diabetes, like mature-onset diabetes of the young. Specifically, triantennary sialylated plasma N-glycan with antennary fucose was very successful in extracting individuals with early-onset diabetes with damaging *HNF1A* mutations.^29^ Our previous studies on N-glycome in T2DM development showed that individuals with a higher risk of disease due to recorded hyperglycemia during acute illness had increased complexity of plasma N-glycome,^30^ possibly reflecting the altered flux of glucose through the hexosamine pathway, which produces uridine diphosphate-N-acetylglucosamine, the substrate for N-linked glycosylation.^24^ Other factors such as presence of monosaccharide-nucleotides or presence of enzymes included in the process of glycosylation are important for its heterogeneity,^17^ as well as the glycan processing pathway through the Golgi apparatus and structural features of the protein part in glycoprotein, especially near the N-glycosylation site, and many more.^31^

Recently, we have also studied glycans as a tool for cardiometabolic risk assessment and proposed the glycan-based score GS_T2D_,^32^ developed on a population with incident T2DM from the European Prospective Investigation into Cancer and Nutrition-Potsdam study. These studies confirmed that plasma N-glycome could be an early predictor of T2DM development. Apart from being associated with various diseases and disorders, plasma N-glycosylation has been thoroughly examined in association with lifestyle factors, such as smoking,^33^ exercise,^34^ sleep^35^ and dietary habits.^36^ While smoking influenced increase in complexity of glycans,^33^ which is associated with a proinflammatory-like glycosylation profile, moderate to vigorous exercise positively affected the plasma glycosylation profile of older female individuals despite them being diagnosed with metabolic syndrome by decreasing levels of triantennary and tetra-antennary glycans,^34^ both hallmarks of complex plasma glycoprofile. Interestingly, higher abundance of biantennary glycan structures with core fucose and one or two sialic acid residues was observed in patients with rapid eye movement sleep behavior disorder when compared with healthy controls.^35^ These simpler, low-branched glycan structures are usually decreased in patients with health issues. One study found dietary habits influencing interesting changes in plasma N-glycosylation.^36^ Increased sialylation was positively associated with a healthier diet, which is opposite to the commonly observed higher abundance of highly sialylated (trisialylated and tetrasialylated) glycans in individuals with various diseases.

Since IR is a known driving factor for T2DM development and its key feature, in addition to previous findings, this study investigated whether plasma N-glycome is predictive of first clinical diagnosis of impaired glucose metabolism, incident IR or T2DM in 534 individuals (89 incident IR/T2DM cases, 445 controls) from the TwinsUK cohort with independent replication. Furthermore, the relationship between N-glycome and time to diagnosis will be explored.

## Research design and methods

### Study population

#### Discovery cohort

**Figure 1:**
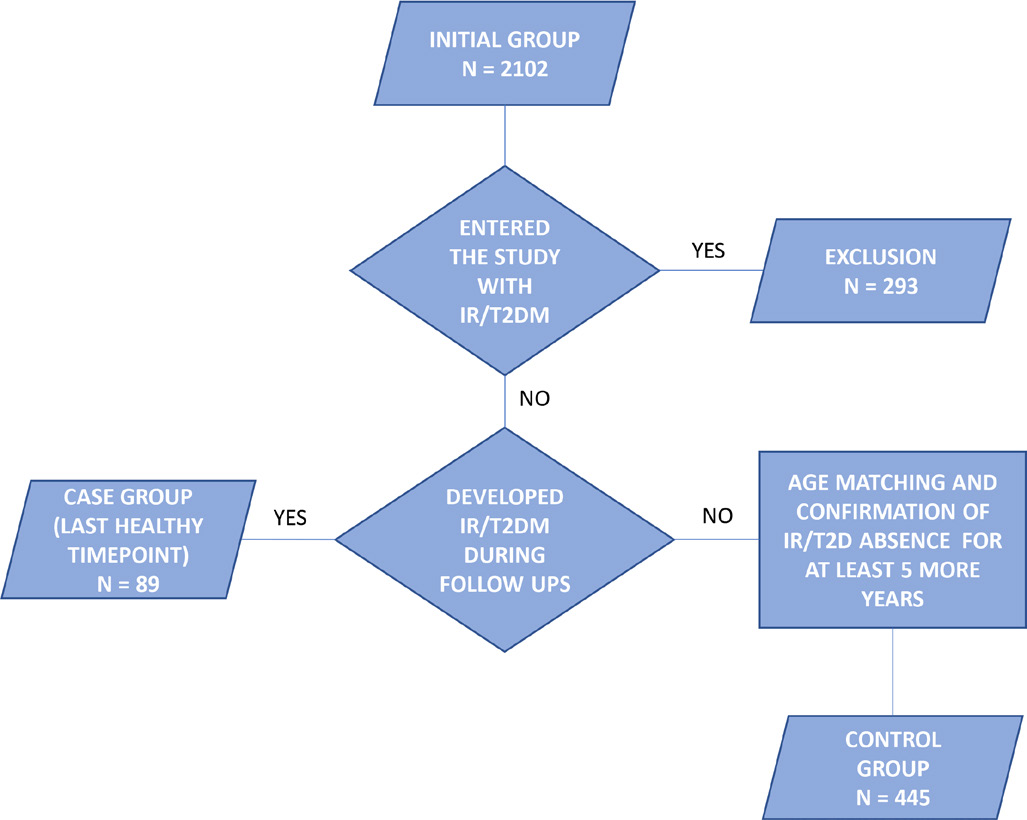
Inclusion flow chart of the TwinsUK cohort. IR, insulin resistance; T2DM, type 2 diabetes mellitus.

We analyzed 6032 plasma samples from the TwinsUK cohort using the hydrophilic interaction ultra-performance liquid chromatography with fluorescence detection (HILIC-UPLC-FLR). The analyzed samples were collected at multiple timepoints throughout a period of 20 years, from 1996 to 2016. Chromatographic profiling of the total plasma protein N-glycome was followed by the glycan data preprocessing protocol (described in detail in the Statistical analysis section), which reduced the glycan data set to 5889 samples. The following statistical analysis was performed on a subset of 534 women, which included incident IR/T2DM cases and independent controls selected from the original set of samples. Case patients were selected based on their first confirmed occurrence of disruption in glucose metabolism—type 2 diabetes (fasting glucose ≥7 mmol/L or physician’s letter confirming diagnosis) or IR (evaluated using homeostatic model assessment (HOMA2) score)—as well as availability of at least one plasma sample taken before the diagnosis. The control group was selected based on a negative type 2 diabetes and IR status during the total follow-up period and only included samples taken at least 5 years before the last visit, in order to exclude patients who will possibly develop the disease in the next 5 years and to obtain truly negative cases. A flow chart of the inclusion and exclusion protocol is depicted in figure 1. Neither the case nor the control group contained siblings. The control group was age-matched with the case group. Description of the cohort is provided in table 1.

**Table 1:** Descriptive statistics of the analyzed T2DM and IR subset of the discovery TwinsUK cohort

Characteristics	TwinsUK cohort (IR/T2DM subset)	
	Cases (IR/T2DM)	Controls
Samples, n	89 (52/47)	445
Female participants, %	100	100
Age, mean±SD, years	55.07±9.01	55.06±8.95
BMI, mean±SD, kg/m^2^	28.15±4.78	25.21±3.99
Follow-up period, mean±SD, years	7.14±3.04	7.14±3.04
Systolic blood pressure, mean±SD, mm Hg	125.73±16.95	122.81±16.51
Diastolic blood pressure, mean±SD, mm Hg	78.86±9.91	76.80±10.12
Active smokers, n (%)	6 (9.4)	15 (5.3)
Non-smokers, n (%)	39 (60.9)	189 (66.3)
Ex-smokers, n (%)	19 (29.7)	81 (28.4)

Data on smoking status were available for 349 participants (65%).

BMI, body mass index; IR, insulin resistance; T2DM, type 2 diabetes mellitus.

#### Replication cohort

As a replication cohort we used samples collected through the Finland Cardiovascular Risk Study (FINRISK) designed to investigate the risk factors for chronic, non-communicable diseases.^37^ We analyzed the samples of 38 participants who developed T2DM (mean age ±SD, years=59.42 ± 8.99) during a 10-year follow-up period and 38 participants who remained unaffected and served as controls (mean age ±SD, years=59.61 ± 8.93).

### Consent to participate

All subjects included in this study gave written, informed consent. All twins in the TwinsUK study provided informed written consent.

### Plasma N-glycome analysis

#### Release of total plasma protein N-glycans

Plasma samples (10 µL) were denatured using 20 µL of 2% (weight/volume (w/v)) sodium dodecyl sulfate (SDS) (Invitrogen, USA), followed by incubation at 65°C for 10 min. Then, 10 µL of 4% (volume/volume (v/v)) Igepal CA-630 (Sigma-Aldrich, St Louis, Missouri, USA) were added to the denatured samples, after which the mixture was shaken for 15 min on a plate shaker (Gujarat Fluorochemicals Limited (GFL), Germany). N-glycans were released by adding 1.2 U of PNGase F (Promega, USA) to the mixture, followed by overnight incubation at 37°C.

#### Labeling and HILIC solid-phase extraction clean-up of released N-glycans

N-glycans released from total plasma proteins were labeled with 2-aminobenzamide (2-AB), a fluorescent dye. The fluorescent labeling mixture was made inhouse from both 2-AB (19.2 mg/mL; Sigma-Aldrich) and 2-picoline borane (44.8 mg/mL; Sigma-Aldrich) dissolved in dimethyl sulfoxide (Sigma-Aldrich) and glacial acetic acid (Merck, Germany) mixture (70:30 v/v). Each sample was labeled by addition of 25 µL of the labeling mixture, followed by 2-hour incubation at 65°C. Following the 2-hour incubation, samples were brought to a total concentration of 96% acetonitrile by addition of 700 µL of 100% cold acetonitrile to each sample. The samples were then transferred to a 0.2 µm hydrophilic polypropylene membrane (GHP) filter plate (Pall Corporation, USA). All wells of the filter plate were prewashed with 70% ethanol (Sigma-Aldrich) and water, followed by equilibration using 96% acetonitrile. Both 70% ethanol and 96% acetonitrile were freshly prepared on the day of the experiment execution. Removal of solvent and impurities from samples, such as excess free labeling dye and reducing agent, was performed by HILIC-solid-phase extraction on the mentioned filter plate with the application of vacuum using a vacuum manifold (Millipore Corporation, USA). Loaded samples were subsequently washed 5× with 96% acetonitrile (ACN). Plasma protein released and labeled N-glycans were finally eluted 2× with 90 µL of ultra-pure water and stored at −20°C until usage.

#### HILIC-UPLC-FLR profiling of total plasma protein released N-glycans

**Figure 2:**
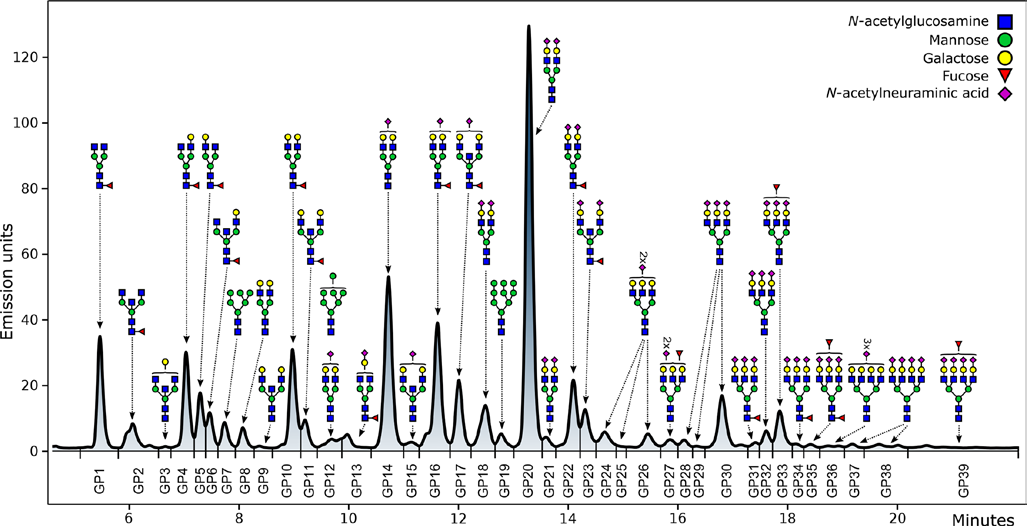
Chromatogram of HILIC-UPLC-FLR-analyzed plasma N-glycome. The most abundant glycan structure is graphically presented for each glycan peak (GP). The y axis represents the intensity of the signal measured in emission units, while retention time in minutes is presented on the x axis. HILIC-UPLC-FLR, hydrophilic interaction ultra-performance liquid chromatography with fluorescence detection.

Fluorescently labeled N-glycans were separated by HILIC on Acquity UPLC H-Class instrument (Waters, Milford, USA) consisting of a quaternary solvent manager, sample manager and a fluorescence detector, set with excitation and emission wavelengths of 250 nm and 428 nm, respectively. The instrument was under the control of Empower V.3 software, build 3471 (Waters). Waters BEH Glycan chromatography column was used to separate labeled N-glycans, with 100 mM ammonium formate, pH 4.4, as solvent A and liquid chromatography-mass spectrometry grade acetonitrile as solvent B. Each 96-well plate contained five standard samples and one blank sample for the purpose of maintaining quality control and performing batch correction. The plasma protein N-glycans separation method used linear gradient of 70%–53% acetonitrile at a flow rate of 0.561 mL/min in a 25 min analytical run. The calibration of the system was done by using an external standard of hydrolyzed and 2-AB labeled glucose oligomers from which the retention times for the individual glycans were converted to glucose units. Automated integration method was used to perform data processing.^38^ The plasma proteins N-glycans chromatograms were all separated equivalently into 39 peaks (GP1–GP39) and are presented in figure 2.

Detailed description of glycan structures corresponding to each glycan peak is presented in online supplemental table 1. Glycan peaks were analyzed based on their elution positions and measured in glucose units. The results were then compared with the reference values from the ‘GlycoStore’ database (available at https://glycostore.org/)^39^ for structure assignment. In addition to 39 directly measured plasma glycan traits, 16 derived traits were calculated. These derived traits represent an average of the particular glycosylation structural feature, like branching, sialylation, galactosylation, fucosylation and incidence of bisecting N-acetylglucosamine (GlcNAc). The formulas used for calculation of plasma protein N-glycan derived traits are presented in online supplemental table 2.

10.1136/bmjdrc-2021-002263.supp1 Supplementary data

### Statistical analysis

#### Data normalization

The raw data represented as areas under each glycan peak had to be normalized in order to remove the experimental noise. This area was divided by the total integrated area and multiplied by 100, expressing each peak as a percentage of the total integrated area. This allows different patients’ glycan peaks to be compared, regardless of absolute intensities obtained by analysis of their samples.

#### Batch correction

To remove the effects of experimental variation during sample preparation and analysis, batch correction was performed on normalized glycan data. The data were first log-transformed due to the right-skewness of its distribution. The experimental noise was then reduced by applying the ComBat method from the R package ‘sva’. Within the model, the corresponding order number of sample plates, representing the source of variation, was set as a batch covariate. This procedure was performed for every glycan peak.

#### Analysis of last prediagnostic timepoint

In order to identify glycan structures which are significantly different in patients prior to diagnosis of T2DM or IR compared with controls, general linear models were used. For each glycan, log-transformed relative area under the glycan peak was modeled as a dependent variable, while disease status was used as an independent variable. Since the control group was age-matched and there were no siblings in either of the groups, no additional covariates were included in the model. This was repeated for each of 39 glycan peaks and 16 derived glycan traits. We adjusted for multiple testing using Benjamini-Hochberg false discovery rate, with adjusted p value of <0.05 considered as significant. Obtained effect sizes represent the natural logarithm of the difference in relative area of the corresponding glycan peak in prediagnostic patients compared with controls.

#### Prediction of disease development

Discriminatory potential of individual glycan peaks was further evaluated using area under the receiver operating characteristic curve (AUC). From the set of eight glycan peaks significantly associated with disease status in the previous step, the best predicting ones were identified using stepwise regression variable selection method. It was carried out in bidirectional approach based on Akaike information criteria to find the best performing model and reduce overfitting. Six glycan peaks selected by stepwise regression along with the body mass index (BMI) data were included in the logistic regression model. Using receiving operator characteristic (ROC) curves, it was compared with BMI data alone, which represented the null model, as well as the model containing only six selected glycan peaks. The ‘pROC’ package was used to construct the ROC curves and calculate the corresponding AUC.

#### Temporal progression of glycan abundances

The dynamics of glycan abundances in the disease development period were also represented. All available case group samples, ranging from 10 years before the diagnosis of IR or T2DM up to 2 years after the diagnosis, were split into four groups (10–8 years, 7–5 years, 4–2 years before and 0–2 years after the diagnosis) and included in the linear mixed effects model. As fixed effects, log-transformed relative area under the glycan peak was modeled as the dependent variable, while the corresponding temporal groups along with age at time of sampling were included as independent variables. Individual patient identification nested within the family identification was modeled as random effect. From the obtained model using the ‘emmeans’ package, the mean of glycan peak relative area was estimated for each temporal group and back-transformed from the log scale. This provided age-corrected data on average abundancy of individual glycan peak, depending on the time to or from diagnosis. The procedure was repeated for each glycan peak previously identified as significantly changed in the case group compared with controls. Estimated means were then graphically represented along with 95% CI.

## Results

### Plasma N-glycome is extensively different in individuals prior to their IR/T2DM diagnosis when compared with unaffected controls

**Figure 3:**
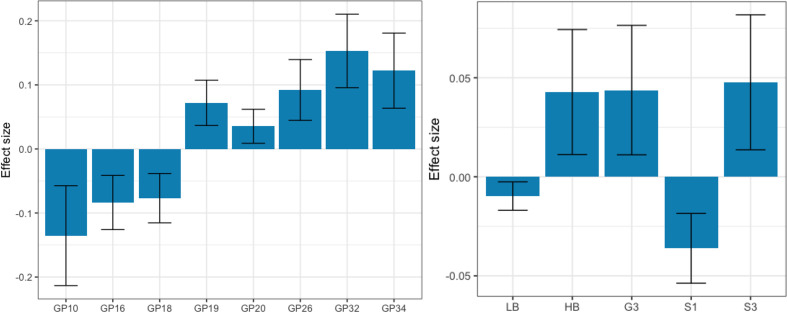
Effects of insulin resistance and type 2 diabetes development on the identified significantly differentiated initial (left) and derived plasma N-glycan traits (right) from the TwinsUK cohort. Calculated effect size (natural logarithm of difference in relative area) of each presented initial or derived trait is shown on the y axis with error bar representing 95% CI, while glycan trait name, initial or derived, is displayed on the x axis. G3, trigalactosylated glycans; GP, glycan peak; HB, high-branched glycans; LB, low-branched glycans; S1, monosialylated glycans; S3, trisialylated glycans.

Using HILIC-UPLC method, we have analyzed and profiled total plasma protein N-glycome in 534 patients from the TwinsUK cohort. Of these, 445 were unaffected individuals who have not developed IR nor T2DM during the study period (controls) and 89 were individuals who were diagnosed with IR/T2DM in one of the timepoints (the IR/T2DM cases). General linear model was used to examine differences in initial and derived plasma glycan traits between the IR/T2DM cases (last sample prior to the disease diagnosis; mean time before diagnosis 4.65±2.36) and the controls. Out of 39 directly measured initial glycan traits, 8 showed statistically significant difference. GP10, GP16 and GP18 were decreased, while GP19, GP20, GP26, GP32 and GP34 were increased, in IR/T2DM cases, as shown in figure 3 and online supplemental table 3.

Besides investigating differences in directly measured initial glycans, we have also searched for significant differences in derived traits, which represent an average of the same glycosylation feature, shared among different glycan structures and are calculated from the initial glycan traits. After adjusting for multiple testing, out of 16 derived plasma N-glycome traits, 5 were significantly altered in affected individuals when compared with the controls: monosialylated (S1) and low-branched N-glycans showed a significant decrease, while high-branched, trigalactosylated (G3) and trisialylated (S3) N-glycans were significantly increased, in IR/T2DM cases (figure 3, online supplemental table 3).

The effects of T2DM and IR development on the identified initial and derived plasma N-glycan traits are depicted separately in online supplemental figure 1.

### Plasma N-glycome continuously changes toward the diagnosis of IR or T2DM

**Figure 4:**
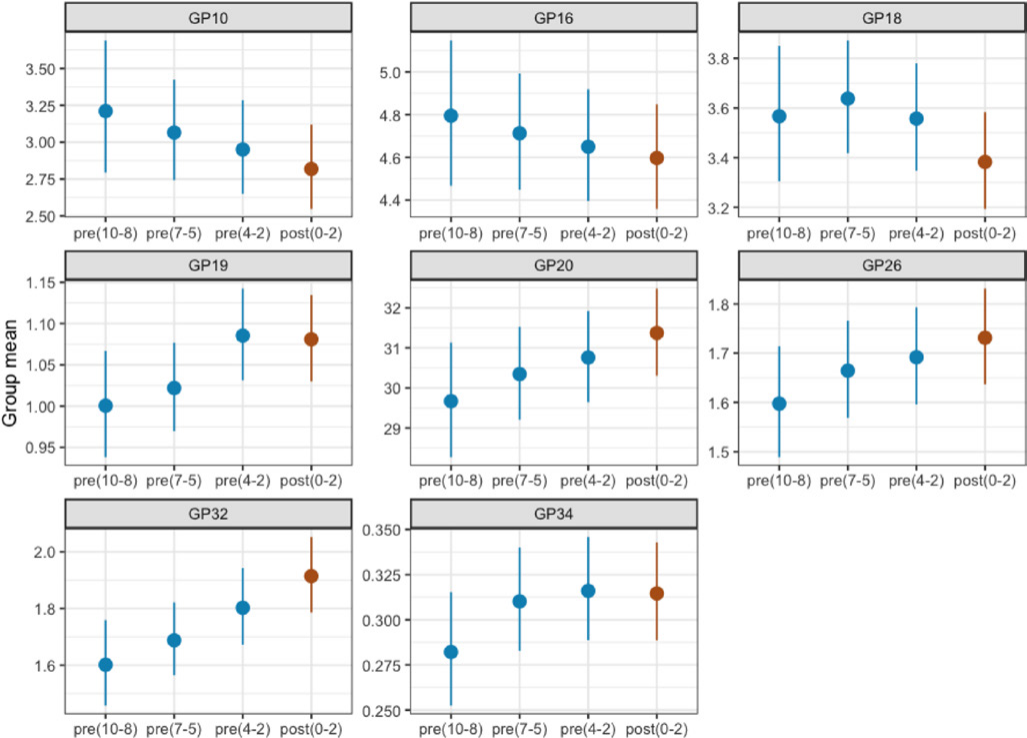
Time-to-diagnosis behavior of the most significantly altered initial glycan traits in individuals who developed insulin resistance/type 2 diabetes from the TwinsUK cohort. Glycan abundance is presented on the y axis with dots representing calculated means and lines representing 95% CI, while different temporal groups are presented on the x axis. The timepoint period in which the disease diagnosis occurred is labeled brown, while all prediagnosis timepoint periods are labeled blue. Numbers listed inside the brackets represent years of distance from diagnosis. GP, glycan peak.

Next, we examined the time-to-diagnosis behavior of eight initial glycan groups that showed predictive potential for IR/T2DM development. Their abundances were plotted through different timepoints from the 10 years prior to disease development to the disease onset (figure 4). Each temporal group consisted of all case samples available for chosen timepoint prior to disease diagnosis; for example, temporal group 8–10 years prior to diagnosis consisted of glycan data analyzed from all available samples taken 8–10 years prior to diagnosis. The groups did not differ in age (the comparison of N-glycan levels with the control group of the corresponding age for each timepoint is shown in online supplemental figure 2, with average age of both cases and controls shown in online supplemental table 4). The differences between temporal groups for GP10, GP16, GP20, GP26 and GP32 showed a continuous trend toward disease development. GP19 even shows an increase in this trend 2–4 years prior to diagnosis, while GP18 displays a change in direction 5–7 years before diagnosis establishment. GP34 shows the largest glycan abundance increase from 8–10 to 5–7 years prior to diagnosis, after which their levels start to stagnate. These results demonstrate that the human plasma N-glycome develops more visible and notable alterations as the diagnosis of either IR or T2DM approaches.

### Stratification of IR/T2DM-prone individuals using N-glycans

**Figure 5:**
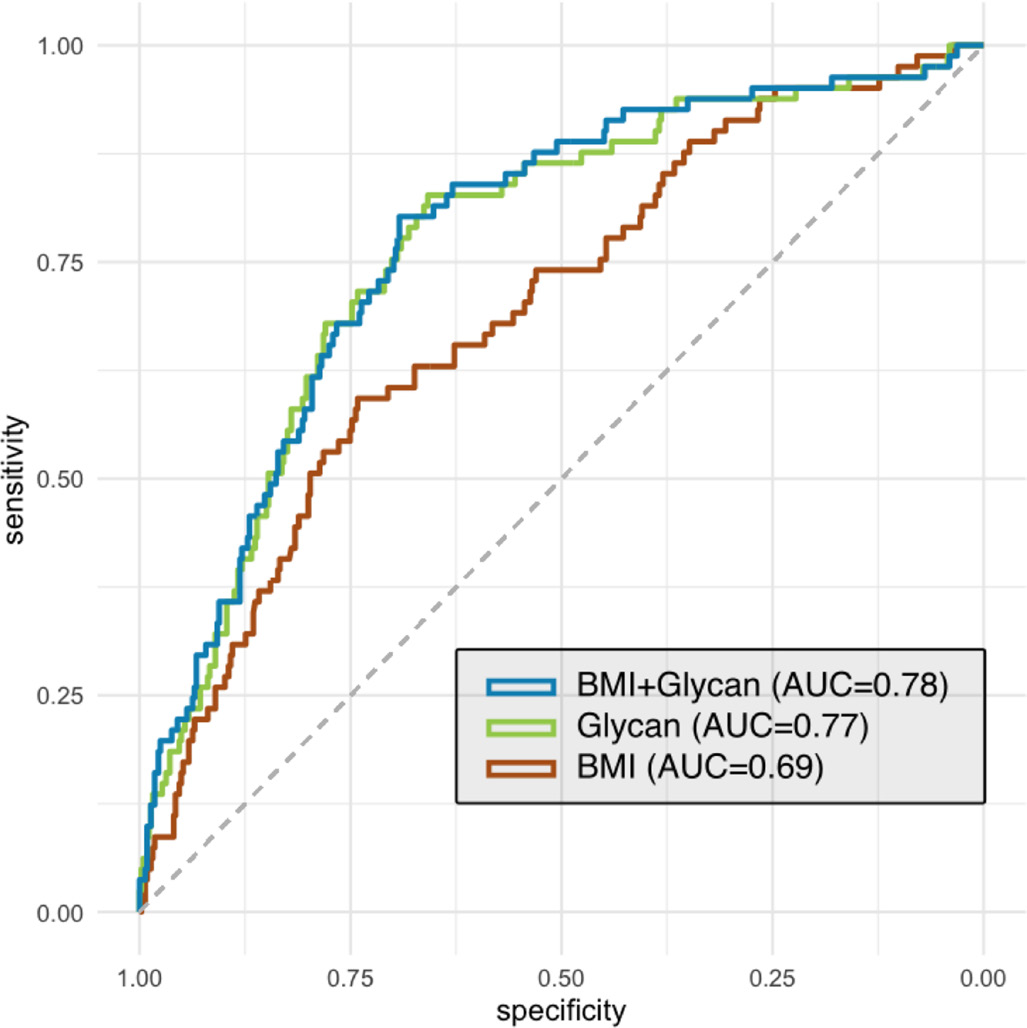
Stratification performance of insulin resistance/type 2 diabetes prediction model created from the TwinsUK cohort data. Comparison of prediction model based on selected glycan peaks with the model including the BMI data alone and the model combining both BMI and glycan data. Dashed grey diagonal line represents no-discrimination reference. AUC, area under the receiver operating characteristic curve; BMI, body mass index.

T2DM prediction and progression models are key factors for diabetes management and could be the source of significant improvements in health status and overall quality of life for the individuals with increased risk for this serious health burden. Therefore, in order to improve the IR/T2DM prediction, we have built a ‘glyco diabetes prediction model’ using the most informative glycan variables (GP10, GP16, GP18, GP19, GP20 and GP34). The model was built from the last available data before disease diagnosis. We further added BMI to the model as it is a known risk factor for IR/T2DM,^40^ thus creating a model combining glycan and BMI information (‘glyco-BMI diabetes prediction model’). The performance of each model was examined using ROC curve analysis. The result of ROC analysis is the graphic representation of the stratification ability based on the comparison of glycan abundances in 534 individuals. The AUC value of ‘glyco diabetes prediction model’ is 0.77, while the AUC of the ‘glyco-BMI diabetes prediction model’ is 0.78, and for the model consisting only of BMI data (*‘*BMI diabetes prediction model’) it is 0.69. These results show that glycans are valuable predictors of IR/T2DM. Furthermore, the fact that BMI has negligible contribution to glycan prediction model indicates that glycans already encompass the information the BMI data can provide (figure 5). Additional data for each point of the ROC curves are provided in online supplemental table 5. We have also compared the predictiveness of glycans with BMI and other risk factor data (smoking status and blood pressure) available only for the portion of TwinsUK cohort participants (online supplemental figure 3).

10.1136/bmjdrc-2021-002263.supp2 Supplementary data

### External validation of significantly altered initial glycan traits on an independent T2DM follow-up cohort (FINRISK)

We replicated our findings in FINRISK, an independent T2DM cohort. We compared baseline plasma protein N-glycan abundances of both 38 incident cases (the ones who developed T2DM during a 10-year-follow-up) and 38 controls. We were able to show the same direction of changes for all previously identified significantly altered initial N-glycans (GP10, GP18, GP19, GP20, GP26, GP32, GP34), except for GP16, with GP32 displaying statistically significant difference between the groups (adjusted p=6.62×10^−03^; online supplemental table 6). Mentioned glycan alterations in FINRISK cohort are depicted in online supplemental figure 4.

## Conclusions

Here we show that plasma N-glycome can identify individuals who will develop either IR or T2DM years before the clinical diagnosis. Individuals healthy at baseline and developing IR/T2DM during the follow-up time have a higher presence of complex, highly branched glycans within their plasma N-glycome, accompanied by a decrease in low-branched structures. Increase in the complexity and branching of the plasma N-glycome is a hallmark of many pathological conditions with inflammatory component,^41^ as well as aging,^21^ and was also observed in previous studies investigating the changes of plasma N-glycome connected to T2DM.^27 30^ It has been suggested that the increased branching of plasma N-glycome actually reflects the disruption in the glucose metabolism through the altered flux of glucose through the hexosamine pathway.^30^ We have previously reported that N-glycans (GS_T2D_ score) could predict T2DM development 6–8 years before the disease onset. Herein we strengthen our result by including not only the individuals developing T2DM, but also individuals developing IR as their first diagnosis of impaired glucose metabolism. Since IR is known as a condition which very commonly precedes the development of T2DM for even a decade,^42 43^ we have now evaluated the predictive potential of N-glycans earlier in life.

The two glycan peaks which showed the most significant alteration in incident IR/T2DM cases in this study were GP32 (p=9.52×10^−06^) and GP34 (p=9.38×10^−04^). GP32 is a structure consisting of a trigalactosylated trisialylated triantennary glycan structure (A3G3S3), while GP34 is the mixture of mostly core fucosylated trigalactosylated trisialylated triantennary glycan structure (FA3G3S3) and less tetragalactosylated trisialylated tetra-antennary glycan structure (A4G4S3). A3G3A3 structure, in plasma, predominantly originates from α1-acid glycoprotein (AGP), while A4G4S3 completely derives from this positive acute phase protein,^44^ whose increased levels were found to be significantly associated with diabetes in many studies.^45–47^ Even in our recent study, which focused on a cardiometabolic risk assessment, GP32 (A3G3S3) was recognized as one of the six structures strongly associated with T2DM development.^32^ Considering these findings, future studies should definitely focus on investigating the N-glycosylation of AGP in diabetes, such as a recently published paper which developed high-throughput method for AGP N-glycosylation analysis and tested it on a population of individuals with hyperglycemia at high risk of T2DM development.^48^

Our examination of time-to-diagnosis behavior of significantly altered glycan structures showed that modification of plasma N-glycome started even 10 years before the clinical presentation and diagnosis of glucose metabolism impairment, either IR or T2DM, and it continuously proceeds throughout the disease onset.

To determine the discriminative power of plasma N-glycans in identifying individuals at risk of IR/T2DM, we have built a ‘glyco diabetes prediction model’ using the most significantly different and informative N-glycan structures. This glyco model had the discriminative power greater than the model with BMI data alone and almost identical as the BMI–glycan combined model. Our ‘glyco diabetes prediction model’ had similar performance as other, non-glycan-based, suggested T2DM prediction models.^49^ Its clinical value could be possibly further improved when combined with other known risk factors, but unfortunately we did not have sufficient data available for our patients to evaluate this potential. Our findings on diabetes prediction using glycans, as well as other similar studies on this subject,^30 31^ definitely point out the importance of using plasma N-glycans in existing and future T2DM prediction models and risk assessment tools since we have shown that glycans encompass the information BMI data carry, but also provide additional predictive value.

Comparison of baseline plasma N-glycans in FINRISK incident diabetes cases with that of healthy controls confirmed our findings on the increase of more complex glycan structures present in plasma N-glycome prior to disease diagnosis. It is important to highlight that in the FINRISK cohort we have compared glycans from individuals’ samples taken 10 years before the T2DM diagnosis occurred, which means that probably some participants in the mean time (baseline timepoint to follow-up timepoint) developed/were diagnosed with IR or pre-diabetes. Therefore, these baseline samples of future T2DM cases could display the earliest noticeable changes in glycans resulting from underlying development of glucose metabolism impairment.

We note some study limitations. First, the results were discovered primarily in women. Second, our replication cohort is quite small-numbered, which may pose as a risk of bias. Third, due to unavailability of various patient clinical data, we were unable to further test glycans’ clinical value by evaluating the discriminative potential of a model composed of glycans and other T2DM risk factors (apart from BMI). Also, it is important to mention that BMI, while widely and most commonly used, is not the best clinical marker for diabetes prediction, especially in older population,^50^ where it has been shown that the association of BMI and diabetes decreases with age. On the other hand, BMI is calculated solely from body weight and height and offers no information regarding body fat percentage, which showed to bring added informative value in identifying individuals at high risk of abnormal glucose levels.^51^ Unfortunately, body fat percentage data were not available for this study. Fourth, in our study we have investigated only the glycan portion of the plasma glycoproteins, and not the protein part. Changes in protein levels,^52^ their clearance rate, structure^53^ and other parameters are known to be influenced by a myriad of various processes, such as different diseases,^54^ lifestyle habits, oxidative status and others,^55^ all of which also heavily influence glycans.^17 21 56^ Therefore, it is important to mention that some of the observed glycan alterations might also be a result of changes in the protein portion of plasma glycoproteins.

In conclusion, our study further highlights the role of T2DM prediction using plasma N-glycome. This opens the possibility of early assessment of individuals at high risk of disrupted glucose metabolism which can lead to prevention or delay of T2DM development, increasing the quality of life and at the same time decreasing treatment costs. We have shown that plasma N-glycome is not only altered years before T2DM development, but also precedes IR diagnosis, showing that the impairment of glycome starts to manifest even earlier than it was previously shown. Taking into consideration this early manifestation, glycome changes are certainly associated with underlying progression of T2DM, which is known to take years to develop and present symptoms.^57 58^ It is still unknown whether these changes are the cause or the consequence of the disease; however, it is now certain that they play an important role in diabetes development and that the change of glycans within a person could warn about possible disease development and allow both the clinician and the patient to take adequate steps to prevent or delay disease development.

## Data Availability

Data are available upon reasonable request. The datasets used and analysed during the current study are available from the corresponding author on reasonable request.
