# Supplementary material for: Plasma N-glycome shows continuous deterioration as the diagnosis of insulin resistance approaches
Source: BMJ Open Diabetes Res Care. 2021 Sep 9;9(1):e002263. doi: 10.1136/bmjdrc-2021-002263 (PMC8438737; doi:10.1136/bmjdrc-2021-002263)
Supplement: Supplementary data [file bmjdrc-2021-002263supp001.pdf]

Supplementary Materials

Plasma N-glycome shows continuous deterioration as the diagnosis of insulin resistance approaches

Ana Cvetko[1]*, Massimo Mangino[2,3]*, Marko Tijardović[1], Domagoj Kifer[1], Mario Falchi[2], Toma Keser[1], Markus Perola[4], Tim D Spector[2], Gordan Lauc[1,5], Cristina Menni[2]*, Olga Gornik[1]*

[1]Faculty of Pharmacy and Biochemistry, University of Zagreb, Zagreb 10000, Croatia

[2]Department of Twin Research and Genetic Epidemiology, King's College London, Westminster Bridge Road, SE17EH London, UK

[3]NIHR Biomedical Research Centre at Guy's and St Thomas' Foundation Trust, London SE1 9RT, UK

[4]National Institute for Health and Welfare, Helsinki, Finland

[5]Genos Glycoscience Research Laboratory, Zagreb 10000, Croatia

*These authors contributed equally

Corresponding author:

Olga Gornik

ogornik@pharma.unizg.hr

Faculty of Pharmacy and Biochemistry, University of Zagreb

A. Kovačića 1, 10 000 Zagreb, Croatia

Supplementary Table 1 Abbreviation and description of all glycan structures complementary to every plasma glycan peak.*

Sample	***Glycan peak***	***Glycan structure abbreviation***	***Glycan structure description***	***Glycan peak abundance formula***
plasma	GP1	FA2	core fucosylated, biantennary	GP1 / GP * 100
plasma	GP2	FA2B; M5	core fucosylated, biantennary with bisecting GlcNAc;<br>oligomannose	GP2 / GP * 100
plasma	GP3	A2BG1	monogalactosylated, biantennary with bisecting GlcNAc	GP3 / GP * 100
plasma	GP4	FA2[6]G1	core fucosylated and monogalactosylated, biantennary	GP4 / GP * 100
plasma	GP5	FA2[3]G1	core fucosylated and monogalactosylated, biantennary	GP5 / GP * 100
plasma	GP6	FA2[6]BG1	core fucosylated and monogalactosylated, biantennary with bisecting GlcNAc	GP6 / GP * 100
plasma	GP7	M6;<br>FA2[3]BG1	oligomannose;<br>core fucosylated and monogalactosylated, biantennary with bisecting GlcNAc	GP7 / GP * 100
plasma	GP8	A2G2	digalactosylated, biantennary	GP8 / GP * 100
plasma	GP9	A2BG2	digalactosylated, biantennary with bisecting GlcNAc	GP9 / GP * 100
plasma	GP10	FA2G2	core fucosylated, digalactosylated, biantennary	GP10 / GP * 100
plasma	GP11	FA2BG2	core fucosylated, digalactosylated, biantennary with bisecting GlcNAc	GP11 / GP * 100
plasma	GP12	M7;<br>A2G2S1;<br>A1M4G1S1;<br>A2BG1S1	oligomannose;<br>digalactosylated, monosialylated, biantennary;<br>tetramannosylated, monogalactosylated, monosialylated, monoantennary;<br>monogalactosylated, monosialylated, biantennary with bisecting GlcNAc	GP12 / GP * 100
plasma	GP13	FA2G1S1;<br>FA2BG1S1	core fucosylated, monogalactosylated and monosialylated biantennary;<br>core fucosylated, monogalactosylated and monosialylated biantennary with bisecting GlcNAc	GP13 / GP * 100
plasma	GP14	A2G2S1	digalactosylated and monosialylated biantennary	GP14 / GP * 100
plasma	GP15	A2BG2S1	digalactosylated and monosialylated biantennary with bisecting GlcNAc	GP15 / GP * 100
plasma	GP16	FA2G2S1	core fucosylated, digalactosylated and monosialylated biantennary	GP16 / GP * 100
plasma	GP17	FA2BG2S1	core fucosylated, digalactosylated and monosialylated biantennary with bisecting GlcNAc	GP17/ GP * 100
plasma	GP18	A2G2S2;<br>FA2G2S2	digalactosylated and disialylated biantennary;<br>core fucosylated, digalactosylated and disialylated biantennary	GP18 / GP * 100

plasma	GP19	M9	oligomannose	GP19 / GP * 100
plasma	GP20	A2G2S2	digalactosylated and disialylated biantennary	GP20 / GP * 100
plasma	GP21	A2G2S2;<br>A3G3S1;<br>FA2G2S2;<br>A2BG2S2;<br>A3F1G3S1	digalactosylated and disialylated biantennary;<br>trigalactosylated and monosialylated triantennary;<br>core fucosylated, digalactosylated and disialylated biantennary;<br>digalactosylated and disialylated biantennary with bisecting GlcNAc;<br>antennary fucosylated, trigalactosylated and monosialylated triantennary	GP21 / GP * 100
plasma	GP22	FA2G2S2	core fucosylated, digalactosylated and disialylated biantennary	GP22 / GP * 100
plasma	GP23	FA2BG2S2	core fucosylated, digalactosylated and disialylated biantennary with bisecting GlcNAc	GP23 / GP * 100
plasma	GP24	A3G3S2;<br>A3F1G3S1	trigalactosylated and disialylated triantennary;<br>antennary fucosylated, trigalactosylated and monosialylated triantennary	GP24 / GP * 100
plasma	GP25	A3G3S2;<br>FA2F1G2S2;<br>A3F1G3S2	trigalactosylated and disialylated triantennary;<br>core fucosylated, antennary fucosylated, digalactosylated and disialylated biantennary;<br>antennary fucosylated, trigalactosylated and disialylated triantennary	GP25 / GP * 100
plasma	GP26	A3G3S2;<br>FA3G3S2	trigalactosylated and disialylated triantennary;<br>core fucosylated, trigalactosylated and disialylated triantennary	GP26 / GP * 100
plasma	GP27	A3F1G3S2;<br>A3G3S3	antennary fucosylated, trigalactosylated and disialylated triantennary;<br>trigalactosylated and trisialylated triantennary	GP27 / GP * 100
plasma	GP28	A3G3S3;<br>A3F1G3S2	trigalactosylated and trisialylated triantennary;<br>antennary fucosylated, trigalactosylated and disialylated triantennary	GP28 / GP * 100
plasma	GP29	A3G3S3;<br>A3F1G3S2	trigalactosylated and trisialylated triantennary;<br>antennary fucosylated, trigalactosylated and disialylated triantennary	GP29 / GP * 100
plasma	GP30	A3G3S3;<br>A3F1G3S3	trigalactosylated and trisialylated triantennary;<br>antennary fucosylated, trigalactosylated and trisialylated triantennary	GP30 / GP * 100
plasma	GP31	FA3G3S3;<br>A3G3S3	core fucosylated, trigalactosylated and trisialylated triantennary;<br>trigalactosylated and trisialylated triantennary	GP31 / GP * 100
plasma	GP32	A3G3S3	trigalactosylated and trisialylated triantennary	GP32 / GP * 100
plasma	GP33	A3F1G3S3	antennary fucosylated, trigalactosylated and trisialylated triantennary	GP33 / GP * 100
plasma	GP34	FA3G3S3;<br>A4G4S3	core fucosylated, trigalactosylated and trisialylated triantennary;<br>tetragalactosylated and trisialylated tetraantennary	GP34 / GP * 100
plasma	GP35	FA3F1G3S3;<br>A4F1G4S3	core fucosylated, antennary fucosylated, trigalactosylated and trisialylated triantennary;<br>antennary fucosylated, tetragalactosylated and trisialylated tetraantennary	GP35 / GP * 100

plasma	GP36	A4G4S3;<br>A4F1G4S3	tetragalactosylated and trisialylated tetraantennary;<br>antennary fucosylated, tetragalactosylated and trisialylated tetraantennary	GP36 / GP * 100
plasma	GP37	A4G4S4;<br>A4F1G4S3	tetragalactosylated and tetrasialylated tetraantennary;<br>antennary fucosylated, tetragalactosylated and trisialylated tetraantennary	GP37 / GP * 100
plasma	GP38	A4G4S4;<br>A4F1G4S4;<br>A4F1G4S3	tetragalactosylated and tetrasialylated tetraantennary;<br>antennary fucosylated, tetragalactosylated and tetrasialylated tetraantennary;<br>antennary fucosylated, tetragalactosylated and trisialylated tetraantennary	GP38 / GP * 100
plasma	GP39	A4F1G4S4;<br>A4F2G4S4	antennary fucosylated, tetragalactosylated and tetrasialylated tetraantennary;<br>antennary difucosylated, tetragalactosylated and tetrasialylated tetraantennary;	GP39 / GP * 100

*structure abbreviations – all N-glycans have two core *N*-acetylglucosamines (GlcNAcs); F at the start of the abbreviation indicates a core-fucose α1,6-linked to the inner GlcNAc; Mx, number (x) of mannose on core GlcNAcs; Ax, number of antenna (GlcNAc) on trimannosyl core; A2, biantennary with both GlcNAcs as β1,2-linked; A3, triantennary with a GlcNAc linked β1,2 to both mannose and the third GlcNAc linked β1,4 to the α1,3 linked mannose; A4, GlcNAcs linked as A3 with additional GlcNAc β1,6 linked to α1,6 mannose; B, bisecting GlcNAc linked β1,4 to β1,3 mannose; G(x), number (x) of β1,4 linked galactose on antenna; F(x), number (x) of fucose linked α1,3 to antenna GlcNAc; S(x), number (x) of sialic acids linked to galactose

Supplementary Table 2 Formulas for plasma N-glycome derived glycan traits calculation. 16 derived traits are calculated out of 39 directly measured initial plasma glycan traits.

Plasma N-glycome derived trait	***Formula***
Low branching (mono- and biantennary glycans) (LB)	GP1+GP2+GP3+GP4+GP5+GP6+GP8+GP9+GP10+GP11+0.5xGP12+GP13+GP14+ GP15+GP16+GP17+GP18+GP20+GP21+GP22+GP23
High branching (tri- and tetraantennary glycans) (HB)	GP24+GP25+GP26+GP27+GP28+GP29+GP30+GP31+GP32+GP33+GP34+ GP35+GP36+GP37+GP38+GP39
Agalactosylation (G0)	GP1+GP2
Monogalactosylation (G1)	GP3+GP4+GP5+GP6+GP13
Digalactosylation (G2)	GP8+GP9+GP10+GP11+0.5xGP12+GP14+GP15+GP16+GP17+GP18+GP20+GP21+GP22+GP23
Trigalactosylation (G3)	GP24+GP25+GP26+GP27+GP28+GP29+GP30+GP31+GP32+GP33+GP34+ GP35
Tetragalactosylation (G4)	GP36+GP37+GP38+GP39
Neutral glycans (S0)	GP1+GP2+GP3+GP4+GP5+GP6+GP8+GP9+GP10+GP11
Monosialylation (S1)	0.5xGP12+GP13+GP14+GP15+GP16+GP17
Disialylation (S2)	GP18+GP20+GP21+GP22+GP23+GP24+GP25+GP26+GP27
Trisialylation (S3)	GP28+GP29+GP30+GP31+GP32+GP33+GP34+GP35+GP36
Tetrasialylation (S4)	GP37+GP38+GP39
Incidence of bisecting GlcNAc (B)	GP2+GP3+GP6+GP9+GP11+GP15+GP17+GP23
Antennary fucosylation (AF)	GP27+GP33+GP35+GP39
Core fucosylation (CF)	GP1+GP2+GP4+GP5+GP6+GP10+GP11+GP13+GP16+GP17+GP22+GP23+GP31+GP34+GP35
Oligomannose glycans (M)	GP7+0.5xGP12+GP19

Supplementary Table 3 The development of IR/T2DM induces significant changes in directly measured plasma N-glycans and plasma N-glycome derived traits in the TwinsUK cohort. Calculated effect size (natural logarithm of difference in relative area) for each of statistically significant derived trait is listed along with corresponding adjusted p-values.

Plasma N-glycome peak	***Effect size***	***95% Confidence interval***	***Adjusted p value***
**GP10**	-0.14	(-0.2134, -0.3488)	3.99 $x10^{-03}$
**GP16**	-0.08	(-0.126, -0.2096)	9.38 $x10^{-04}$
**GP18**	-0.08	(-0.1154, -0.1922)	9.38 $x10^{-04}$
**GP19**	0.07	(0.0366, 0.1086)	9.38 $x10^{-04}$
**GP20**	0.04	(0.0089, 0.0443)	4.48 $x10^{-02}$
**GP26**	0.09	(0.0446, 0.1367)	1.05 $x10^{-03}$
**GP32**	0.15	(0.0957, 0.2487)	9.52 $x10^{-06}$
**GP34**	0.12	(0.0636, 0.1859)	9.38 $x10^{-04}$
Plasma N-glycome derived trait	***Effect size***	***95% Confidence interval***	***Adjusted p value***
**LB**	-0.01	(-0.0169, -0.0266)	2.84 $x10^{-02}$
**HB**	0.04	(0.0113, 0.0541)	2.84 $x10^{-02}$
**G3**	0.04	(0.0111, 0.0549)	2.84 $x10^{-02}$
**S1**	-0.04	(-0.0537, -0.0899)	1.07 $x10^{-03}$
**S3**	0.05	(0.0136, 0.0614)	2.84 $x10^{-02}$

Supplementary Table 4 Average age of both cases and controls for each temporal group created around the diagnosis.

Temporal group according to disease diagnosis	***cases*** *age (mean ± SD), years*	***controls*** *age (mean ± SD), years*	***case*** *samples, N*	***control*** *samples, N*
pre 8-10 years	53.35 ± 8.91	53.36 ± 8.97	23	115
pre 5-7 years	54.78 ± 8.40	54.77 ± 8.40	37	185
pre 2-4 years	53.98 ± 8.36	53.72 ± 8.05	40	198
post 0-2 years	59.69 ± 8.35	59.02 ± 7.60	49	237

Supplementary Table 5 ROC curve point data showing specificity, sensitivity, Youden's index, true neutrals (tn), true positives (tp), false negatives (n) and false positives (fp) for each cut-off threshold of the three tested models. Data for each model is listed on a separate document sheet. Available as a separate file.

Supplementary Table 6 The development of T2DM induces significant changes in directly measured plasma N-glycans in the FINRISK cohort. Calculated effect size (natural logarithm of difference in relative area) for each of statistically significant derived trait is listed along with corresponding adjusted p-values.

Plasma N-glycome peak	***Effect size***	***95% Confidence interval***	***Adjusted p value***
**GP10**	-0.33	(-0.8355, 0.1159)	5.92×10^{-01}
**GP16**	0.06	(-0.3139, 0.4017)	7.68×10^{-01}
**GP18**	-0.24	(-0.4553, -0.0325)	1.47×10^{-01}
**GP19**	0.04	(-0.0215, 0.0990)	5.92×10^{-01}
**GP20**	0.55	(-0.4580, 1.6736)	5.92×10^{-01}
**GP26**	0.14	(-0.0061, 0.2606)	3.02×10^{-01}
**GP32**	0.24	(0.1072, 0.3757)	6.62×10^{-03}
**GP34**	0.04	(0.0078, 0.0728)	7.83×10^{-02}

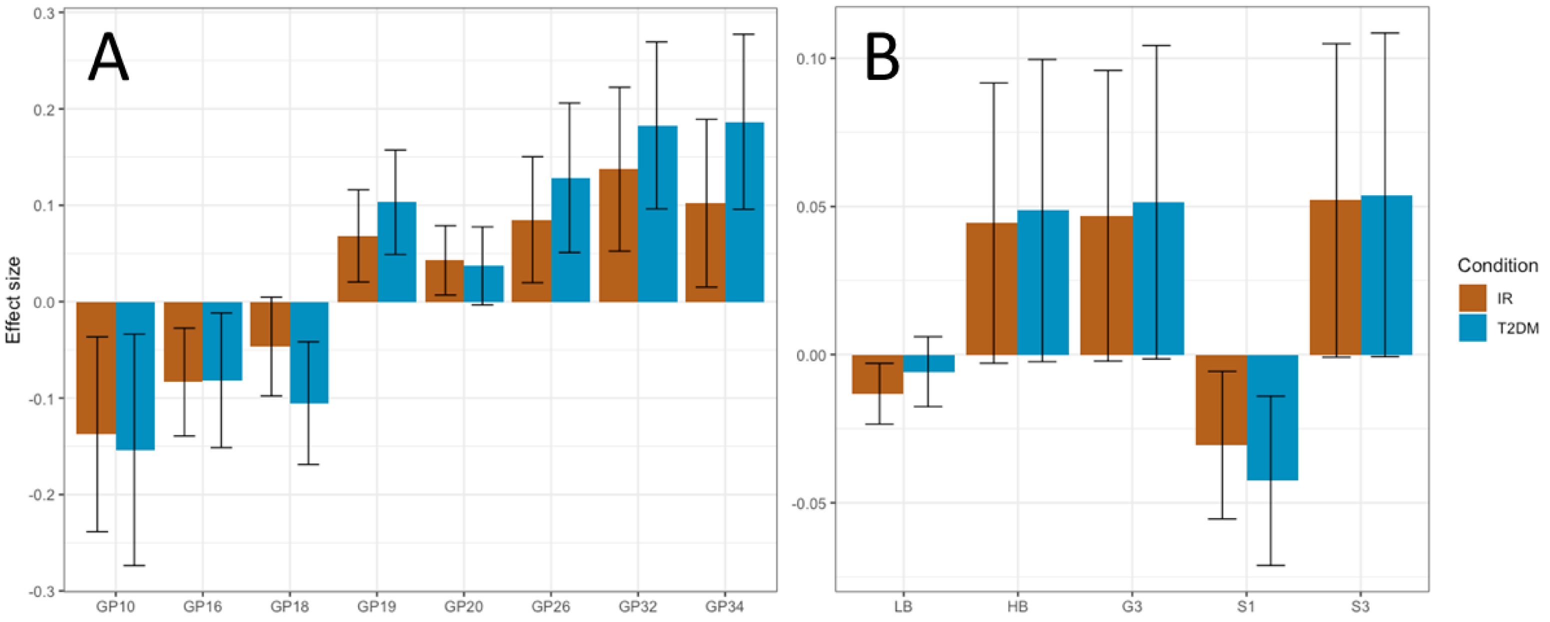

Supplementary Figure 1 Individual effects of T2DM and IR development on the identified significantly differentiated initial (A) and derived plasma N-glycan traits (B) from the TwinsUK cohort. Calculated effect size (natural logarithm of difference in relative area) of each presented initial or derived trait is shown on the y axis with error bar representing the 95% confidence interval, while glycan trait name is displayed on the x axis. (GP – glycan peak, LB – low branched glycans, HB – high branched glycans, G3 – trigalactosylated glycans, S1 – monosialylated glycans, S3 – trisialylated glycans)

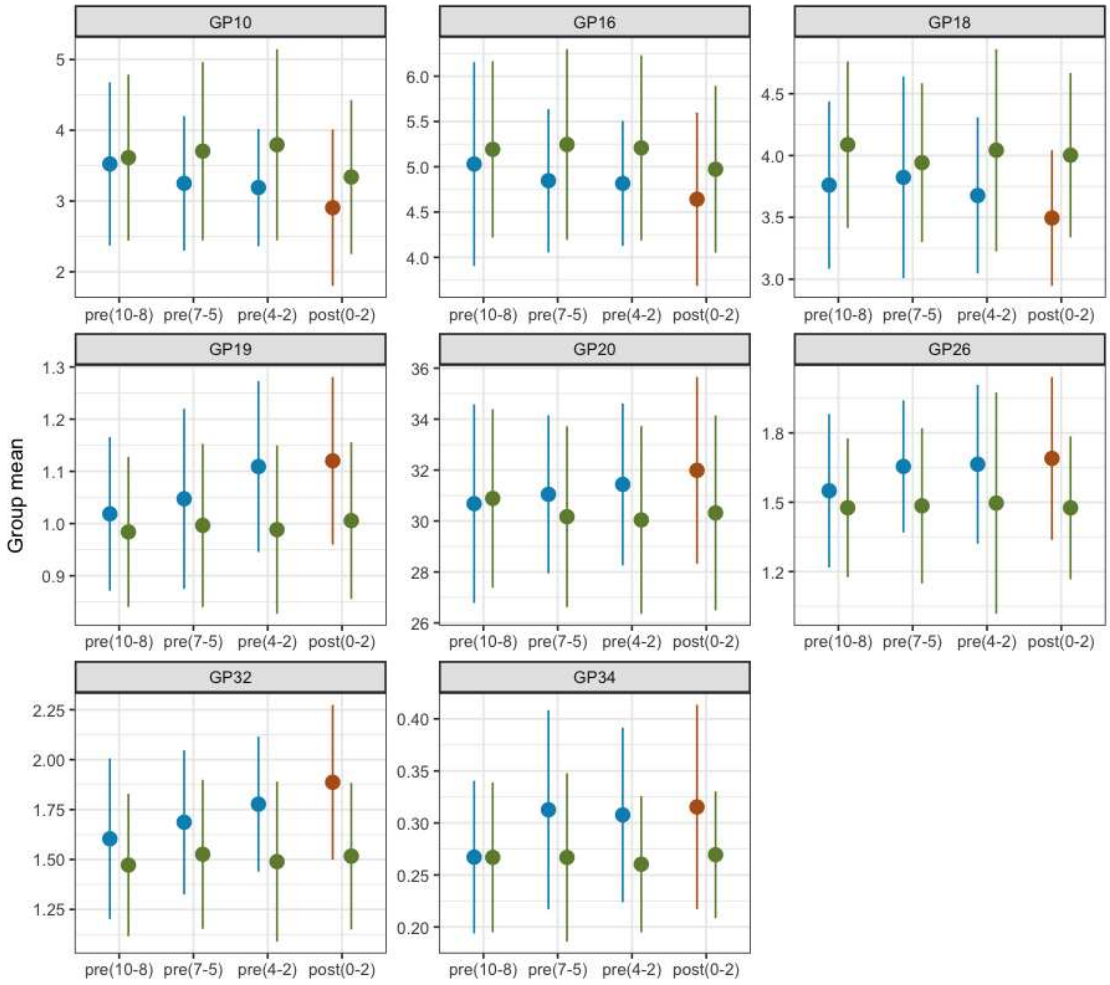

Supplementary Figure 2 Time-to-diagnosis behaviour of the most significantly altered initial glycan traits in individuals who developed IR/T2DM compared with the control group of the corresponding age for each timepoint from the TwinsUK cohort. Glycan abundances are presented on the y axis with dots representing calculated means and lines representing 95% confidence intervals, while different temporal groups are presented on the x axis. The timepoint period in which the disease diagnosis occurred is labelled brown, while all pre-diagnosis timepoint periods are labelled blue. Glycan levels of control groups are labelled green. Numbers listed inside the brackets represent years of distance from diagnosis.

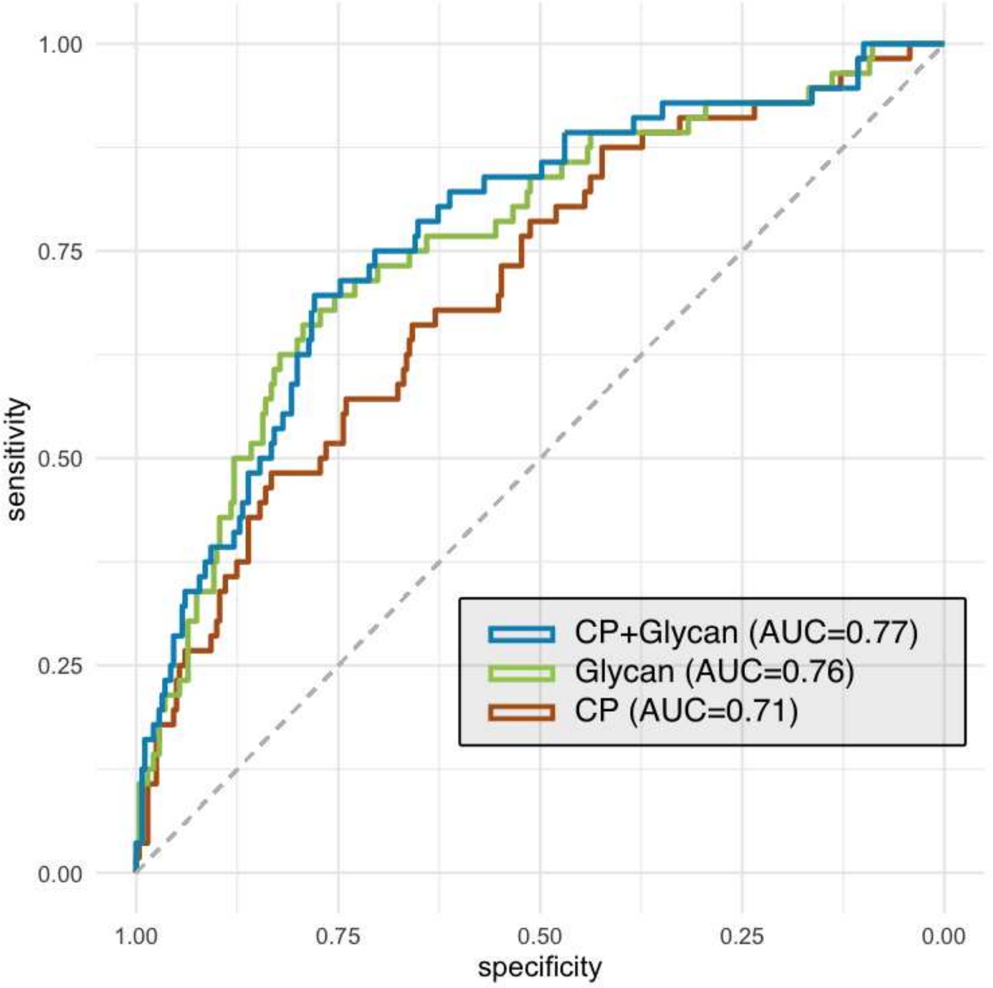

Supplementary Figure 3 Stratification performance of IR/type 2 diabetes prediction models created from the available TwinsUK cohort data. Comparison of prediction model based on glycan peaks independently associated with IR/type 2 diabetes (labelled green) with the model including the BMI, smoking status and blood pressure data alone (grouped as risk factors, labelled brown) and the model combining both mentioned risk factors and glycan data (labelled blue).

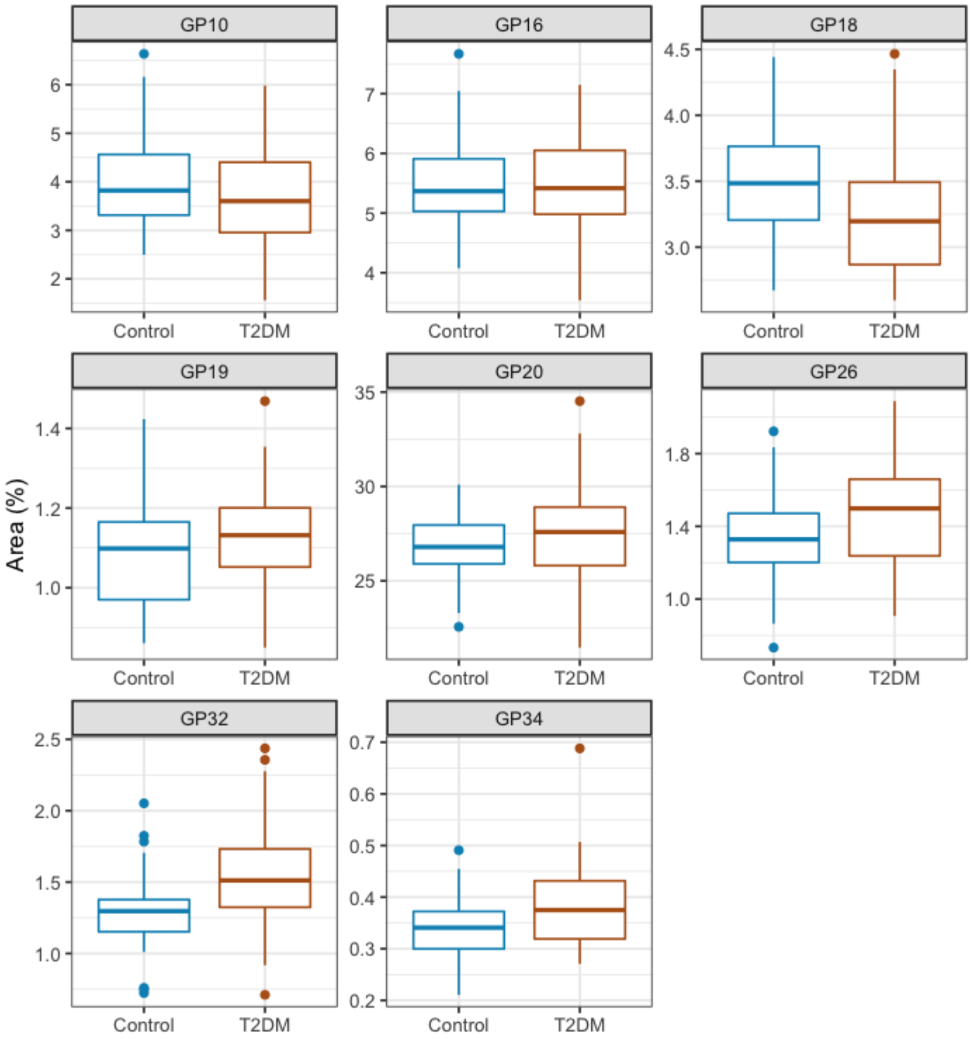

Supplementary Figure 4 Significant plasma protein N-glycan alterations externally validated on FINRISK confirmation cohort displayed with boxplots. Y axis represents the glycan abundances of FINRISK participants at baseline sampling. Participants who developed T2DM during follow-up are labelled brown. Participants who remained unaffected at follow-up sampling are labelled blue. Boxes in the boxplot are ranging from 25th to 75th percentile with the median represented as a line inside the box. The whiskers of the boxplot extend from both percentiles, 75th percentile for upper whisker and 25th for lower whisker, to the values within 1.5xIQR. IQR is the inter-quartile range, the distance from the first to the third quartile. Data outside the whiskers' ends are outliers and are plotted individually.
